# Multi-Reader Multi-Case Study for Performance Evaluation of High-Risk Thyroid Ultrasound with Computer-Aided Detection

**DOI:** 10.3390/cancers12020373

**Published:** 2020-02-06

**Authors:** Ming-Hsun Wu, Kuen-Yuan Chen, Shyang-Rong Shih, Ming-Chih Ho, Hao-Chih Tai, King-Jen Chang, Argon Chen, Chiung-Nien Chen

**Affiliations:** 1 Department of Surgery, National Taiwan University Hospital, Taipei 10002, Taiwan; 010028@ntuh.gov.tw (M.-H.W.); dtsurg51@ntuh.gov.tw (K.-Y.C.); mcho1215@ntu.edu.tw (M.-C.H.); taihc1@ntu.edu.tw (H.-C.T.); kingjen@ntu.edu.tw (K.-J.C.); 2 Department of Internal Medicine, National Taiwan University, Taipei 10002, Taiwan; srshih@ntu.edu.tw; 3 Graduate Institute of Industrial Engineering, National Taiwan University, Taipei 10617, Taiwan

**Keywords:** ultrasonography, thyroid nodule, thyroid cancer, computer-aided detection

## Abstract

Physicians use sonographic characteristics as a reference for the possible diagnosis of thyroid cancers. The purpose of this study was to investigate whether physicians were more effective in their tentative diagnosis based on the information provided by a computer-aided detection (CAD) system. A computer compared software-defined and physician-adjusted tumor loci. A multicenter, multireader, and multicase (MRMC) study was designed to compare clinician performance without and with the use of CAD. Interobserver variability was also analyzed. Excellent, satisfactory, and poor segmentations were observed in 25.3%, 58.9%, and 15.8% of nodules, respectively. There were 200 patients with 265 nodules in the study set. Nineteen physicians scored the malignancy potential of the nodules. The average area under the curve (AUC) of all readers was 0.728 without CAD and significantly increased to 0.792 with CAD. The average standard deviation of the malignant potential score significantly decreased from 18.97 to 16.29. The mean malignant potential score significantly decreased from 35.01 to 31.24 for benign cases. With the CAD system, an additional 7.6% of malignant nodules would be suggested for further evaluation, and biopsy would not be recommended for an additional 10.8% of benign nodules. The results demonstrated that applying a CAD system would improve clinicians’ interpretations and lessen the variability in diagnosis. However, more studies are needed to explore the use of the CAD system in an actual ultrasound diagnostic situation where much more benign thyroid nodules would be seen.

## 1. Introduction

Thyroid nodules are very common findings. The clinical importance of thyroid nodules lies primarily with the possibility of thyroid cancer, which occurs in approximately 8–15% of all thyroid nodules [1]. Accurate initial thyroid nodule work-up is key to identifying clinically significant thyroid cancer, and new diagnostic tools, such as Fourier transform infrared (FTIR) and Raman spectroscopy, are sought to complement the present medical procedures [2,3,4].

Advances in high-resolution ultrasonography (US) have led to an increased availability of information on thyroid nodules and to efficient and effective diagnosis of patients with malignant thyroid nodules [1]. US is useful for not only detection of but also discrimination between benign and malignant lesions and is used as guidance for fine-needle aspiration (FNA) and surgical intervention [5,6]. There are a large number of studies about the role of grayscale (B-mode) US in the diagnosis of malignant thyroid nodules [7,8,9,10,11], and important features, such as microcalcification, hypoechogenicity, and taller than wide, are used in the prediction of thyroid malignancy according to the 2015 American Thyroid Association Guideline Task Force [6] and American College of Radiology Thyroid Imaging Reporting and Data System (ACR TI-RADS) [12]. Physicians identify sonographic characteristics as a reference for the possibility of diagnosing thyroid cancers and the need for further examination or treatment in clinical practice. Therefore, the presence of suspicious sonographic characteristics may serve as indicators for the malignancy potential of thyroid nodules. However, US is very subjective and highly dependent on the skill of the performer, and it is criticized for its possible inter- and intraobserver variability [13,14,15,16,17,18]. Unnecessary FNA and even diagnostic surgery are common following US findings [19]. The proposal of US-based malignancy risk stratification systems [6,12] may improve interobserver agreements [20] but would still be heavily dependent on the operator’s ability to accurately describe key nodule features.

Computerized quantification methods can be used to characterize the sonographic features and make the diagnosis more objective [21,22,23,24], and a computer-aided detection (CAD) system has been developed and cleared by the US FDA (K180006) for clinical use. The purpose of this study was to investigate whether physicians were more effective in their tentative diagnosis of thyroid cancer (confidence level of the malignancy potential) based on the presence of sonographic characteristics with the “information” provided by CAD. A multicenter, multireader, and multicase (MRMC) study was therefore designed to compare clinician performance with and without CAD for the detection of malignant thyroid nodules.

## 2. Materials and Methods

### 2.1. Study Design

The projected total numbers of readers and cases were obtained to ensure a test power of 0.8 based on the Dorfman–Berbaum–Metz (DBM) method for MRMC test studies [25,26] and a pilot study dataset with 7 readers and 130 images of thyroid nodules (73 benign and 57 malignant). Accordingly, the reader study was performed with a sufficient sample size of ultrasound images and a sufficient number of physicians. This reader study thus recruited 19 medical professionals (readers) to read 265 thyroid ultrasound images based on initial investigations of AUROC increase and random effects from readers and cases. The physicians were recruited from multiple centers, including National Taiwan University Hospital, National Cheng Kung University Hospital, Chang Gung Memorial Hospital, Changhua Christian Hospital, Lukang Christian Hospital, China Medical University Hospital, Kaohsiung Veterans General Hospital, Taipei Municipal Wanfang Hospital, Tri-Service General Hospital, Taipei Municipal Gan-Dau Hospital, and Good Liver Clinic. All readers were board-certified specialists in Endocrine & Metabolism, Otolaryngology, or Family Medicine with 2–31 years’ experience in Thyroid diagnostic ultrasound. They have interpreted more than 400 thyroid ultrasound images per year for an average of 9.47 (range, 2 to 31) years. None of them collected the images in the study cases. Moreover, neither the readers nor the CAD system had foreknowledge of the pathology results.

The institutional review board of National Taiwan University Hospital, which provided the sonogram images, approved the study (200805039R). Patients in the study signed informed consent for the use of their data/images. All procedures performed in the studies involving human participants were in accordance with the ethical standards of the institutional and/or national research committee and with the 1964 Helsinki declaration and its later amendments or comparable ethical standards.

A total of 280 ultrasound images of thyroid nodules were collected as a case pool (15 in the exercise set and 265 in study set) To demonstrate the device applicability across all levels of ultrasound imaging technologies, these cases acquired from three different ultrasound scanners. There were 50 cases collected from an HDI 5000 (Philips Medical Systems, Bothell, WA, USA), 105 cases collected from a Voluson 730 (GE Medical Systems, Milwaukee, WI, USA), and 125 cases collected from an ALOKA Prosound 2 (Hitachi Medical Systems Europe, Zoetermeer, The Netherlands). Ultrasound examinations were performed by three board-certified physicians (MH Wu, KY Chen, and SR Shih) with experience of more than 20 years. The images were captured as still images. Image analysis was conducted offline using the DICOM format of images on a separate computer. All of these cases consisted of ultrasound examination images and surgical pathology examinations collected retrospectively from diagnostic thyroid nodule evaluations at National Taiwan University Hospital (NTUH) from March 2009 to July 2013, excluding those nodules with sizes larger than the probe (5.2 cm) multinodular goiters without a separable nodule on ultrasound images. The cases of thyroid diseases were diverse, representing the actual clinical practice and demographic distribution of the patient population. The majority ethnic group was Chinese. It is reported in the literature that there should be no difference between different ethnic groups in thyroid cancer sonographic characteristics [27,28,29,30,31].

In summary, there were 200 patients with 265 nodules in the study set (265 images). Among the 265 nodules, 165 were pathology-proven benign nodules, and 100 were pathology-proven malignant nodules. Of the benign nodules, 139 (84.2%) were in females and 26 (15.8%) were in males. Of the malignant nodules, 79 (79%) were in females and 21 (21%) were in males. The mean age was 46.8 years (in a range of 20.4–84.8 years) for the participants. The mean age was 48.1 years for those with benign nodules and was 44.8 years (in a range of 1.5–84.8 years) for those with malignant nodules. It has been reported that as people ages the prevalence of clinically relevant thyroid nodules increases while the risk of nodule malignancy decreases [32]. In this study, the patient age was not used as a feature for differentiation of malignant nodules and was not at all revealed to the readers of ultrasound images. Among the 165 benign nodules, there were 134 nodular hyperplasia and 31 follicular adenomas. Among the 100 malignant nodules, there were 94 papillary thyroid carcinomas, four follicular carcinomas, one medullary carcinoma, and one poorly differentiated thyroid carcinoma. There were more than 4 board-certified pathologists involved in diagnostic work-up of the histology in the study. Reports were generated mainly by one pathologist and double confirmed by at least 2 pathologists. Nodules with definite histology results were evaluated and analyzed irrespective of the patients.

Three benign and two malignant thyroid sonograms from each ultrasound system were selected for the exercise set. That is, 15 images were used to help readers become familiarized with the scoring definitions, and the cases in the exercise set were relatively typical benign or malignant nodules selected among the images of the case pool. Before the reading sessions, all the information in the study set and exercise set that could identify the patient’s identity were hidden or removed.

Image analysis was conducted off-line using the DICOM image format on a separate computer. First, the operators provided the long and short axes, i.e., the 4 end points of the axes on the margin, of the thyroid nodule. Based on the given axis end points, the CAD software (AmCAD-UT, AmCad BioMed, Taiwan) calculated the contour of the mass to distinguish it from normal thyroid tissue, the so-called semiautomated technique for tumor segmentation. Three different clinicians (MH Wu, KY Chen, and SR Shih) with >20 years of experience in performing US thyroid examinations together evaluated the quality of the nodule segmentation provided by the CAD system and adjusted the locus if needed by consensus. None of the reviewers had any information regarding a patient’s clinical results. In other words, readers were blinded to the actual pathological results of the nodules to diminish the possible learning effect. Nodule segmentation was performed once per nodule.

**Figure 1:**
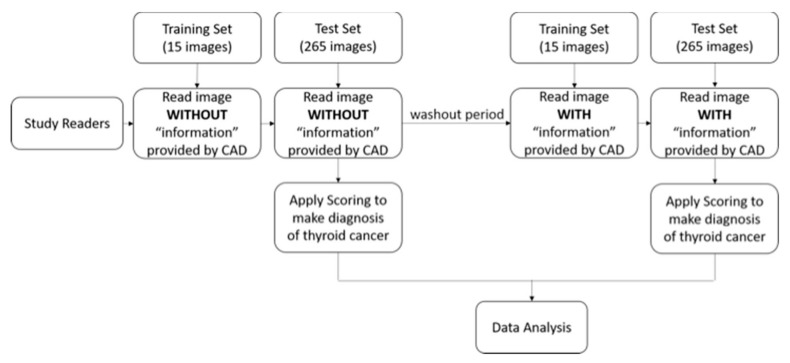
The flowchart for evaluating the reader performance testing of computer-aided detection (CAD).

In the image analysis session, US findings of the segmented mass, including composition (solid or cystic), margins (well-defined or ill-defined), echogenicity (hyperechoic/isoechoic or hypoechoic/marked hypoechoic), texture (heterogeneous or homogeneous), and the presence of echogenic dots suggestive of calcifications were quantified into computerized values by commercial software (AmCAD-UT, AmCad BioMed, Taiwan) and presented as features to describe the thyroid nodule. A flowchart for evaluating the reader performance with or without the CAD results is shown in Figure 1. Briefly, the readers read the same thyroid nodule images twice. The first read was without CAD. To reduce potential reading bias, the second read with CAD was performed after at least two weeks to wash out readers’ memory of the cases [33]. It was shown that the washout time length has no effect on the readers’ performance study in terms of AUROC differences. For both sessions, the readers were given brief training with the exercise set (15 cases) without or with CAD first and subsequent feedback on the pathology results. Then, they interpreted the study set (265 cases). The readers were instructed to give a malignancy probability score to each nodule on a scale of 0 to 100, where a score of 0 would indicate that all similar-looking nodules would be benign according to the observer and a score of 100 would indicate that all similar-looking nodules would be malignant according to the observer. The observers assessed the malignancy score by visually analyzing nodule morphology. Judgment was left to the readers’ discretion, and no specific criteria for malignancy were provided. This method has been well adapted by many previous MRMC studies to compare the readers’ performances [34,35].

### 2.2. Analysis

A computer compared software-defined and physician-adjusted tumor loci. If, for a given point on the physician-adjusted tumor locus, the distance to the nearest point on the software-defined locus was larger than 1 mm, the point was said to be mismatched. Otherwise, the point was matched. All points on the physician-adjusted tumor locus were compared, and the ratio of the number of matched points to the total number of points was calculated and referred to as the match ratio. The match ratio was classified into three categories: (1) Excellent match, referring to a match ratio of 100%; (2) satisfactory match, referring to a match ratio above 70%; and (3) poor match, referring to a match ratio below 70%. The first two categories (excellent and satisfactory categories) were regarded as successful nodule segmentation, as mentioned in the previous study [36].

For statistical analysis of reading performance, MRMC receiver operating characteristic (ROC) analysis was performed. A proper binormal model was used to create the ROC curves. Areas under the ROC curves (AUCs) without and with use of CAD were compared. Differences in observer performance were tested using the Dorfman, Berbaum and Metz method (DBM MRMC package), which takes into account the random effects of the cases, readers, reading modalities and their interactions [37]. In addition, the partial AUC (pAUC) at a false positive interval between 0 and 0.3, corresponding to a specificity ranging from 70% to 100%, were computed. From these ROC curves, individual readers’ sensitivities were calculated at a specificity of 95%. The individual readers’ specificities were also calculated at a sensitivity of 95%. Differences between the paired AUROCs obtained without and with CAD with a test *p*-value less than 0.05 were considered statistically significant.

## 3. Results

### 3.1. Quality of CAD Nodule Segmentation

Excellent, satisfactory, and poor segmentations were observed in 25.3% (*n* = 67), 58.9% (*n* = 156), and 15.8% (*n* = 42) of the nodules, in in 22.4% (*n* = 37), 59.4% (*n* = 98), and 18.2% (*n* = 30) of the benign nodules, and in 30% (*n* = 30), 58% (*n* = 58), and 12% (*n *= 12) of the malignant nodules, respectively. Successful nodule segmentation was significantly more frequent for malignant thyroid nodules than for benign thyroid nodules (*p* = 0.006) (Table 1).

### 3.2. Performance of Observers Without and With CAD

**Figure 2:**
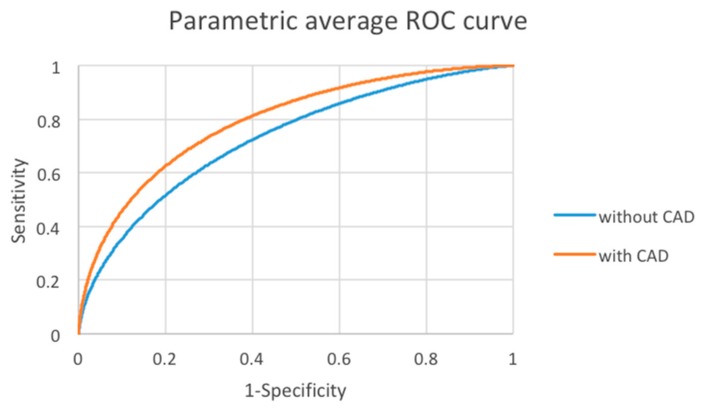
Average area under the curve (AUC) without and with CAD of all readers. Red = with CAD; Blue = without CAD.

The areas under the curve (AUCs) of each reader without and with CAD are shown in Table 2. All readers had an increased performance, 8 of whom showed significant increases, with the use of CAD. The average AUCs of all readers with and without CAD are shown in Table 3. The area under the curve was 0.728 without CAD and significantly increased to 0.792 with CAD (*p *< 0.0001) (Figure 2). This demonstrates that reading thyroid nodule sonograms with the assistance of CAD increased the overall interpretation performance of the readers.

**Figure 3:**
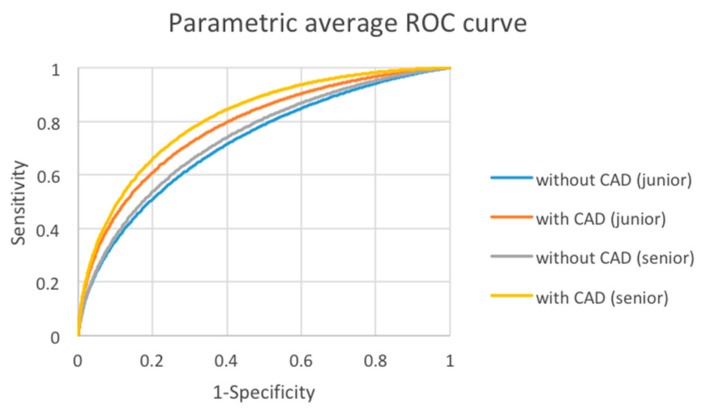
Average AUC Without and With CAD of junior group and senior group. Blue = junior group without CAD; Orange = junior group with CAD; Gray = senior group without CAD; Yellow = senior group with CAD.

The performance of senior and junior observers was further evaluated. The 19 readers were separated into junior and senior groups by their experiences in years on reading thyroid ultrasound images. The experience of the junior group (12 readers), defined as readers with less than 10 years of experience, ranged from 2 to 9 years, and that of the senior group (7 readers), defined as readers with more than 10 years of experience, ranged from 14 to 31 years. The average AUC of the junior group improved from 0.722 to 0.781 with CAD at a statistically significant level (*p* = 0.0059). For the senior group, the average AUC was improved from 0.739 to 0.812 with CAD at a statistically significant level (*p *= 0.0025) (Table 4 and Figure 3). In summary, the reading performance was increased by CAD assistance for both junior and senior groups. There was no significant difference in the improvement between senior and junior clinicians.

### 3.3. Interobserver Variability Analysis

The average standard deviation of the malignant potential score (265 images) among the 19 readers significantly decreased from 18.97 (without CAD) to 16.29 (with CAD) (*p *< 0.0001). The decreases in standard deviation were statistically significant for both benign and malignant cases (18.88 vs. 16.20; *p *< 0.0001 and 19.10 vs. 16.38; *p *= 0.0001) (Table 5).

The mean malignant potential score evaluated by the 19 readers did not significantly change (from 54.92 to 55.14 with *p *= 0.9196) for malignant cases and significantly decreased from 35.01 to 31.24 (*p *= 0.0074) for benign cases (Table 6).

This indicated that with the aid of CAD, the interobserver variability of scoring significantly decreased, and the readers were more confident with their tentative diagnosis, especially for benign cases.

### 3.4. Observer Performance: Sensitivity and Specificity

The partial AUC was estimated in the high specificity portion (0.7–1.0) in Table 7. The pAUC in the high specificity portion was 0.127 without CAD and significantly increased (*p *< 0.0001) to 0.155 with CAD. The average specificity at a sensitivity of 0.95 significantly increased (*p *= 0.0005) from 0.196 to 0.304 with CAD (Table 8). The average sensitivity at a specificity of 0.95 significantly increased (*p *= 0.0022) from 0.252 to 0.328 with CAD (Table 9).

The results showed that with the CAD system, an additional 7.6% of malignant nodules would be recommended to be further evaluated at a specificity of 0.95, and an additional 10.8% of benign nodules would be recommended to be spared from undergoing biopsy at a sensitivity of 0.95.

## 4. Discussion

In this study, a clinical assessment was performed to test the diagnostic performance of a commercial CAD system for the evaluation of thyroid nodules on US images. First, the quality of nodule segmentation was shown to be satisfactory. Second, CAD assistance was shown to increase the interpretation performance of clinical readers. Third, the interobserver variability was decreased with CAD, and the readers were more confident with their tentative diagnoses, especially for benign cases. Fourth, with the aid of the CAD system, at the same level of specificity, clinicians may have detected more malignant nodules to undergo further FNA. Similarly, at the same level of sensitivity, clinicians, with the aid of CAD, may have spared more benign nodules from unnecessary FNA.

Ultrasound is becoming more widely used in screening thyroid nodules. It is the primary diagnostic tool used for assessing the malignancy risk of thyroid nodules and aiding in decision-making regarding the use of FNA [6,38]. The interobserver agreement is the weakness in ultrasound imaging because of its operator dependence and subjective interpretations of its images. ACR TI-RADS recommends the management of thyroid nodules based on their size and the ultrasound features [12]. However, there are many studies of observer variability in thyroid US features leading to inconsistent management [8,13,16,18,39,40]. The CAD system, using artificial intelligence, was expected to improve the diagnostic performance of US and decrease interobserver variability and therefore have the potential to be used as an accompaniment in imaging diagnosis [21,22,23,24,41].

In this study, successful nodule segmentation was observed in 84.2% of nodules with excellent and satisfactory segmentations and was significantly more frequent with malignant thyroid nodules than with benign thyroid nodules (*p *= 0.006). This is consistent with the previous study results and demonstrated that poor segmentation of the thyroid US CAD system may occur and that observer modification is required to reduce improper feature calculation and identification [36]. The results also reflect that in clinical situations, the border of the nodule would be better differentiated and interpreted by a larger difference in echogenicity, which is why more hypoechoic malignant nodules are better segmented by the CAD system. Future technical improvements to the segmentation would be of substantial benefit in saving time/costs for generating reports. In addition, in the study, the evaluation of nodule segmentation quality was conducted by quantitative assessment with the benefit of reducing the subjective bias of qualitative assessment by clinicians, as in other studies [36,42,43]. This CAD format also has the advantage of allowing a third party to validate the results and to assess whether the segmentation chosen for evaluation was adequate.

Several studies have indicated inconsistency in ultrasonography feature interpretation up to 70% [15,18,39]. With the increase in the use of ultrasound images for screening, there is a pressing need for a specialized workflow to reduce the differences in feature interpretation and the reader experience gap while increasing reading accuracy. The results herein indicate that the use of CAD improved the diagnostic performance of the readers in the study in terms of the AUCs. The results of the study also showed that with the aid of CAD, clinician reading is more confident. However, for the malignant probability scores, some observers may use only the lower end of the range between 0 and 100, while others used a more widespread distribution. It should also be noted that a direct comparison of the scores may also overestimate some of the improvements seen. The ROC statistical analysis we used sufficiently considered the relative distribution of the scores and therefore allowed for comparing the performances of the observers.

The previous study compared the CAD system with an experienced radiologist and demonstrated that the CAD system had lower specificity and accuracy [36]. However, the study included only one experienced radiologist from a single center, it might not have reflected the overall clinical setting, and the results might not be generalizable. CAD should act as an assistant instead of a final decision maker, and the benefit brought by the CAD to the clinicians is of primary concern. The present study evaluated whether CAD assistance improves the clinicians’ ability to interpret ultrasound images and aids in the clinical decision. In addition, it showed that the diagnostic accuracy was higher with the help of CAD regardless of the experiences of the users. It had been previously assumed that the senior clinicians might have better diagnostic performance than the junior clinicians [15,17] and thus may benefit less from CAD. This study included observers with various levels of experience to cover diverse clinical situations. The results showed improvements in both the senior and junior clinicians by using CAD, but the improvement was not significantly different between the two groups, which is not consistent with previous studies [15,39,41]. This result may be because most observers recruited in this study were endocrinologists with at least 2 years of experience, and even the clinicians in the junior group already had rich experience in operating and reporting thyroid ultrasound in daily practice.

Ideally, the clinical performance of an imaging technique would be reported to be high if it has high sensitivity and, at the same time, high specificity. More precisely, a higher specificity allows clinicians to reduce the number of misclassified benign lesions, i.e., a lower false positive rate. Likewise, a higher sensitivity allows clinicians to reduce the number of misclassified malignant lesions, i.e., a lower false negative rate. With CAD assistance, the results showed that the added information by CAD might improve the clinician’s biopsy recommendation to prevent 10.8% more benign nodules from unnecessary FNA, while 7.6% more malignant nodules would be suggested for FNA with the aid of CAD. Therefore, the CAD system may be useful as a decision-support tool to detect malignant nodules more accurately and ultimately to avoid unnecessary FNA.

There were several limitations in the current study. First, the observers did not operate or evaluate the real-time US themselves and only interpreted the static images that were selected by a single investigator. Thus, the observers could not take advantage of certain real-time US features. Second, only thyroid nodules > 1.0 cm were included in this study, and selection bias may have been present. Only thyroid nodules with final diagnoses confirmed by surgical specimens were included, which may have led to a higher-than-average nodule malignancy rate. Thus, it cannot demonstrate how many more patients would have been recommended to undergo FNA (or operation) among those who did not have an operation during this study period because they were not biopsied or the FNA was benign. In this situation, we may not know the rate of false positives of the CAD system, and the true sensitivity and specificity cannot be precisely calculated for the general population. More studies are needed to evaluate the performance of the CAD tool for a general population with a greater majority of benign nodules in an actual situation of diagnostic ultrasound imaging. And there are other possible randomized designs for the MRMC study. Third, a high percentage of PTCs (97.4%) with a relatively low percentage of FTCs and FVPTCs may have influenced the diagnostic performance.

## 5. Conclusions

In summary, this MRMC study demonstrated that applying a commercial CAD system would improve clinicians’ interpretive performance and lessen interobserver variability. Therefore, CAD may play an important role in diagnostic decisions for thyroid nodules in the future.

## Figures and Tables

**Table 1:** Quality of CAD system nodule segmentation.

No. of Matches	Excellent (%)	Satisfactory (%)	Poor (%)
All	67 (25.3%)	156 (58.9%)	42 (15.8%)
Benign	37 (22.4%)	98 (59.4%)	30 (18.2%)
Malignancy	30 (30%)	58 (58%)	12 (12%)
*p*-value *	0.006		

* The *p*-value is calculated for the difference in the match ratios between benign and malignant nodules with successful nodule segmentation, which includes excellent and satisfactory cases.

**Table 2:** Reader performance. Area under the ROC curve (AUC) for individual readers without and with CAD.

Reader ID	AUROC		*p*-Value
	Without CAD	With CAD	
Reader 1	0.703	0.776	0.0233
Reader 2	0.728	0.814	0.0004
Reader 3	0.617	0.695	0.5538
Reader 4	0.706	0.765	0.1471
Reader 5	0.673	0.825	<0.0001
Reader 6	0.777	0.820	0.1737
Reader 7	0.793	0.815	0.2197
Reader 8	0.766	0.774	0.7525
Reader 9	0.715	0.819	0.0001
Reader 10	0.771	0.809	0.1209
Reader 11	0.734	0.817	0.0021
Reader 12	0.741	0.773	0.2704
Reader 13	0.753	0.804	0.0664
Reader 14	0.757	0.767	0.7296
Reader 15	0.773	0.824	0.1045
Reader 16	0.742	0.793	0.0326
Reader 17	0.677	0.741	0.2194
Reader 18	0.639	0.807	<0.0001
Reader 19	0.763	0.820	0.0249

**Table 3:** The average AUROC of the readers.

Average	Without CAD (CI)	With CAD (CI)	Difference (CI)	*p*-Value
AUROC	0.728 (0.679, 0.776)	0.792 (0.751, 0.834)	0.065 (0.037,0.092)	<0.0001

Difference = With CAD - Without CAD; CI = 95% Confidence Interval.

**Table 4:** The average AUROC of the junior group and the senior group.

AUROC	Without CAD (CI)	With CAD (CI)	Difference (CI)	*p*-Value
Junior	0.722 (0.666, 0.777)	0.781 (0.737, 0.825)	0.059 (0.025,0.094)	0.0012
Senior	0.739 (0.690, 0.787)	0.812 (0.772, 0.853)	0.074 (0.032,0.115)	0.0025
*p*-value	0.65	0.016		

Difference = With CAD - Without CAD; CI = 95% Confidence Interval.

**Table 5:** The average standard deviation of the malignant potential score among the 19 readers.

Cases	Without CAD (CI)	With CAD (CI)	Difference (CI)	*p*-Value
All	18.97 (18.38, 19.47)	16.29 (15.69, 16.95)	2.38 (1.70, 3.07)	<0.0001
Benign	18.88 (18.20, 19.67)	16.20 (15.54, 16.97)	2.45 (1.62, 3.30)	<0.0001
Malignancy	19.10 (17.88, 19.86)	16.38 (15.65, 17.86)	2.26 (1.14, 3.44)	0.0001

Difference = Without CAD - With CAD; CI = 95% Confidence Interval.

**Table 6:** The mean malignant potential score from the 19 readers.

Cases	Without CAD	With CAD	*p*-Value
Benign	35.01	31.24	0.0074
Malignancy	54.92	55.14	0.9196

**Table 7:** The partial AUC in a specificity range of [0.7, 1.0] from all readers.

Estimate	Without CAD (CI)	With CAD (CI)	Difference (CI)	*p*-Value
pAUC	0.127 (0.106, 0.147)	0.155 (0.133, 0.176)	0.028 (0.015,0.040)	<0.0001

Difference = With CAD - Without CAD; CI = 95% Confidence Interval.

**Table 8:** The average specificity at a sensitivity = 0.95 for all readers.

Average	Without CAD (CI)	With CAD (CI)	Difference (CI)	*p*-Value
Specificity	0.196 (0.121, 0.271)	0.304 (0.211, 0.396)	0.108 (0.050,0.166)	0.0005

Difference = With CAD - Without CAD; CI = 95% Confidence Interval.

**Table 9:** The average sensitivity at a specificity = 0.95 for all readers.

Average	Without CAD (CI)	With CAD (CI)	Difference (CI)	*p*-Value
Sensitivity	0.252 (0.186, 0.319)	0.328 (0.247, 0.409)	0.076 (0.027,0.124)	0.0022

Difference = With CAD - Without CAD; CI = 95% Confidence Interval.

## References

[1] Cooper D.S., Doherty G.M., Haugen B.R., Kloos R.T., Lee S.L., Mandel S.J., Mazzaferri E.L., McIver B., Pacini F., American Thyroid Association Guidelines Taskforce on Thyroid Nodules and Differentiated Thyroid Cancer (2009). Revised American Thyroid Association management guidelines for patients with thyroid nodules and differentiated thyroid cancer. Thyroid.

[2] Rau J.V., Fosca M., Graziani V., Taffon C., Rocchia M., Caricato M., Pozzilli P., Onetti Muda A., Crescenzi A. (2017). Proof-of-concept Raman spectroscopy study aimed to differentiate thyroid follicular patterned lesions. Sci. Rep..

[3] Duraipandian S., Zheng W., Ng J., Low J.J., Ilancheran A., Huang Z. (2013). Near-infrared-excited confocal Raman spectroscopy advances in vivo diagnosis of cervical precancer. J. Biomed. Opt..

[4] Depciuch J., Stanek-Widera A., Skrzypiec D., Lange D., Biskup-Fruzynska M., Kiper K., Stanek-Tarkowska J., Kula M., Cebulski J. (2019). Spectroscopic identification of benign (follicular adenoma) and cancerous lesions (follicular thyroid carcinoma) in thyroid tissues. J. Pharm. Biomed. Anal..

[5] Gharib H., Papini E., Paschke R., Duick D.S., Valcavi R., Hegedus L., Vitti P., AACE/AME/ETA Task Force on Thyroid Nodules (2010). American Association of Clinical Endocrinologists, Associazione Medici Endocrinologi, and European Thyroid Association medical guidelines for clinical practice for the diagnosis and management of thyroid nodules: Executive summary of recommendations. J. Endocrinol. Investig..

[6] Haugen B.R., Alexander E.K., Bible K.C., Doherty G.M., Mandel S.J., Nikiforov Y.E., Pacini F., Randolph G.W., Sawka A.M., Schlumberger M. (2016). 2015 American Thyroid Association Management Guidelines for Adult Patients with Thyroid Nodules and Differentiated Thyroid Cancer: The American Thyroid Association Guidelines Task Force on Thyroid Nodules and Differentiated Thyroid Cancer. Thyroid.

[7] Peccin S., de Castsro J.A., Furlanetto T.W., Furtado A.P., Brasil B.A., Czepielewski M.A. (2002). Ultrasonography: Is it useful in the diagnosis of cancer in thyroid nodules?. J. Endocrinol. Investig..

[8] Wienke J.R., Chong W.K., Fielding J.R., Zou K.H., Mittelstaedt C.A. (2003). Sonographic features of benign thyroid nodules: Interobserver reliability and overlap with malignancy. J. Ultrasound Med..

[9] Iannuccilli J.D., Cronan J.J., Monchik J.M. (2004). Risk for malignancy of thyroid nodules as assessed by sonographic criteria: The need for biopsy. J. Ultrasound Med..

[10] Frates M.C., Benson C.B., Charboneau J.W., Cibas E.S., Clark O.H., Coleman B.G., Cronan J.J., Doubilet P.M., Evans D.B., Goellner J.R. (2005). Management of thyroid nodules detected at US: Society of Radiologists in Ultrasound consensus conference statement. Radiology.

[11] Frates M.C., Benson C.B., Doubilet P.M., Kunreuther E., Contreras M., Cibas E.S., Orcutt J., Moore F.D., Larsen P.R., Marqusee E. (2006). Prevalence and distribution of carcinoma in patients with solitary and multiple thyroid nodules on sonography. J. Clin. Endocrinol. Metab..

[12] Tessler F.N., Middleton W.D., Grant E.G., Hoang J.K., Berland L.L., Teefey S.A., Cronan J.J., Beland M.D., Desser T.S., Frates M.C. (2017). ACR Thyroid Imaging, Reporting and Data System (TI-RADS): White Paper of the ACR TI-RADS Committee. J. Am. Coll. Radiol..

[13] Slapa R.Z., Slowinska-Srzednicka J., Szopinski K.T., Jakubowski W. (2006). Gray-scale three-dimensional sonography of thyroid nodules: Feasibility of the method and preliminary studies. Eur. Radiol..

[14] Park S.H., Kim S.J., Kim E.K., Kim M.J., Son E.J., Kwak J.Y. (2009). Interobserver agreement in assessing the sonographic and elastographic features of malignant thyroid nodules. Am. J. Roentgenol..

[15] Choi S.H., Kim E.K., Kwak J.Y., Kim M.J., Son E.J. (2010). Interobserver and intraobserver variations in ultrasound assessment of thyroid nodules. Thyroid.

[16] Park C.S., Kim S.H., Jung S.L., Kang B.J., Kim J.Y., Choi J.J., Sung M.S., Yim H.W., Jeong S.H. (2010). Observer variability in the sonographic evaluation of thyroid nodules. J. Clin. Ultrasound.

[17] Kim H.G., Kwak J.Y., Kim E.K., Choi S.H., Moon H.J. (2012). Man to man training: Can it help improve the diagnostic performances and interobserver variabilities of thyroid ultrasonography in residents?. Eur. J. Radiol..

[18] Hoang J.K., Middleton W.D., Farjat A.E., Teefey S.A., Abinanti N., Boschini F.J., Bronner A.J., Dahiya N., Hertzberg B.S., Newman J.R. (2018). Interobserver Variability of Sonographic Features Used in the American College of Radiology Thyroid Imaging Reporting and Data System. Am. J. Roentgenol..

[19] Tee Y.Y., Lowe A.J., Brand C.A., Judson R.T. (2007). Fine-needle aspiration may miss a third of all malignancy in palpable thyroid nodules: A comprehensive literature review. Ann. Surg..

[20] Grani G., Lamartina L., Cantisani V., Maranghi M., Lucia P., Durante C. (2018). Interobserver agreement of various thyroid imaging reporting and data systems. Endocr Connect.

[21] Chen K.Y., Chen C.N., Wu M.H., Ho M.C., Tai H.C., Huang W.C., Chung Y.C., Chen A., Chang K.J. (2011). Computerized detection and quantification of microcalcifications in thyroid nodules. Ultrasound Med. Biol..

[22] Wu M.H., Chen C.N., Chen K.Y., Ho M.C., Tai H.C., Chung Y.C., Lo C.P., Chen A., Chang K.J. (2013). Quantitative analysis of dynamic power Doppler sonograms for patients with thyroid nodules. Ultrasound Med. Biol..

[23] Chen K.Y., Chen C.N., Wu M.H., Ho M.C., Tai H.C., Kuo W.H., Huang W.C., Wang Y.H., Chen A., Chang K.J. (2014). Computerized quantification of ultrasonic heterogeneity in thyroid nodules. Ultrasound Med. Biol..

[24] Wu M.H., Chen C.N., Chen K.Y., Ho M.C., Tai H.C., Wang Y.H., Chen A., Chang K.J. (2016). Quantitative analysis of echogenicity for patients with thyroid nodules. Sci. Rep..

[25] Hillis S.L., Berbaum K.S. (2004). Power estimation for the Dorfman-Berbaum-Metz method. Acad. Radiol..

[26] Cesana B.M., Antonelli P., Chiumello D. (2008). Statistical methods for evidence-based medicine: The diagnostic test. Part I. Minerva Anestesiol..

[27] Wong K.T., Ahuja A.T. (2005). Ultrasound of thyroid cancer. Cancer Imaging.

[28] Frates M.C., Benson C.B., Charboneau J.W., Cibas E.S., Clark O.H., Coleman B.G., Cronan J.J., Doubilet P.M., Evans D.B., Goellner J.R. (2006). Management of thyroid nodules detected at US: Society of Radiologists in Ultrasound consensus conference statement. Ultrasound Q..

[29] Yuan W.H., Chiou H.J., Chou Y.H., Hsu H.C., Tiu C.M., Cheng C.Y., Lee C.H. (2006). Gray-scale and color Doppler ultrasonographic manifestations of papillary thyroid carcinoma: Analysis of 51 cases. Clin. Imaging.

[30] Cappelli C., Castellano M., Pirola I., Cumetti D., Agosti B., Gandossi E., Agabiti Rosei E. (2007). The predictive value of ultrasound findings in the management of thyroid nodules. Mon. J. Assoc. Physicians.

[31] Bonavita J.A., Mayo J., Babb J., Bennett G., Oweity T., Macari M., Yee J. (2009). Pattern recognition of benign nodules at ultrasound of the thyroid: Which nodules can be left alone?. Am. J. Roentgenol..

[32] Kwong N., Medici M., Angell T.E., Liu X., Marqusee E., Cibas E.S., Krane J.F., Barletta J.A., Kim M.I., Larsen P.R. (2015). The Influence of Patient Age on Thyroid Nodule Formation, Multinodularity, and Thyroid Cancer Risk. J. Clin. Endocrinol. Metab..

[33] Beiden S.V., Wagner R.F., Doi K., Nishikawa R.M., Freedman M., Lo S.C., Xu X.W. (2002). Independent versus sequential reading in ROC studies of computer-assist modalities: Analysis of components of variance. Acad. Radiol..

[34] Horsch K., Giger M.L., Vyborny C.J., Venta L.A. (2004). Performance of computer-aided diagnosis in the interpretation of lesions on breast sonography. Acad. Radiol..

[35] Van Riel S.J., Ciompi F., Winkler Wille M.M., Dirksen A., Lam S., Scholten E.T., Rossi S.E., Sverzellati N., Naqibullah M., Wittenberg R. (2017). Malignancy risk estimation of pulmonary nodules in screening CTs: Comparison between a computer model and human observers. PLoS ONE.

[36] Choi Y.J., Baek J.H., Park H.S., Shim W.H., Kim T.Y., Shong Y.K., Lee J.H. (2017). A Computer-Aided Diagnosis System Using Artificial Intelligence for the Diagnosis and Characterization of Thyroid Nodules on Ultrasound: Initial Clinical Assessment. Thyroid.

[37] Roe C.A., Metz C.E. (1997). Dorfman-Berbaum-Metz method for statistical analysis of multireader, multimodality receiver operating characteristic data: Validation with computer simulation. Acad. Radiol..

[38] Shin J.H., Baek J.H., Chung J., Ha E.J., Kim J.H., Lee Y.H., Lim H.K., Moon W.J., Na D.G., Park J.S. (2016). Ultrasonography Diagnosis and Imaging-Based Management of Thyroid Nodules: Revised Korean Society of Thyroid Radiology Consensus Statement and Recommendations. Korean J. Radiol..

[39] Brauer V.F., Eder P., Miehle K., Wiesner T.D., Hasenclever H., Paschke R. (2005). Interobserver variation for ultrasound determination of thyroid nodule volumes. Thyroid.

[40] Moon W.J., Jung S.L., Lee J.H., Na D.G., Baek J.H., Lee Y.H., Kim J., Kim H.S., Byun J.S., Lee D.H. (2008). Benign and malignant thyroid nodules: US differentiation--multicenter retrospective study. Radiology.

[41] Jeong E.Y., Kim H.L., Ha E.J., Park S.Y., Cho Y.J., Han M. (2018). Computer-aided diagnosis system for thyroid nodules on ultrasonography: Diagnostic performance and reproducibility based on the experience level of operators. Eur. Radiol..

[42] De Hoop B., Gietema H., van Ginneken B., Zanen P., Groenewegen G., Prokop M. (2009). A comparison of six software packages for evaluation of solid lung nodules using semi-automated volumetry: What is the minimum increase in size to detect growth in repeated CT examinations. Eur. Radiol..

[43] Kim H., Park C.M., Lee S.M., Lee H.J., Goo J.M. (2013). A comparison of two commercial volumetry software programs in the analysis of pulmonary ground-glass nodules: Segmentation capability and measurement accuracy. Korean J. Radiol..

